# Supplementary material for: Census Tract Food Tweets and Chronic Disease Outcomes in the U.S., 2015–2018
Source: Int J Environ Res Public Health. 2019 Mar 18;16(6):975. doi: 10.3390/ijerph16060975 (PMC6466014; doi:10.3390/ijerph16060975)
Supplement: Supplementary file 1 [file ijerph-16-00975-s001.pdf]

SUPPLEMENTAL MATERIALS

Census Tract Food Tweets and Chronic Disease Outcomes in the U.S., 2015-2018

Table S1. Twitter-derived food environment characteristics by state, United States 2015-2018.

State	Total Tweets	Average Caloric Density	Percent of Food Tweets that Mention a Healthy Food	Percent of Food Tweets that Mention Fast Food
AL	8025	248	14.21	15.12
AR	3421	261	16.52	12.80
AZ	17,743	245	13.71	14.11
CA	151,633	253	16.45	10.37
CO	18,766	235	12.26	10.99
CT	11,442	251	14.55	14.97
DC	8469	247	18.01	8.78
DE	2795	250	17.57	15.21
FL	74,393	242	15.22	10.12
GA	28,875	253	17.69	12.01
IA	6928	219	13.31	11.30
ID	3137	259	12.85	23.33
IL	38,482	256	13.93	11.95
IN	14,137	256	13.85	15.05
KS	5390	245	14.03	15.27
KY	8168	254	12.43	21.57
LA	13,159	239	14.13	9.21
MA	25,434	246	14.82	9.09
MD	17,731	237	14.59	13.03
ME	3499	225	16.98	5.69
MI	21,348	243	11.72	18.94
MN	12,634	234	13.70	8.01
MO	14,601	249	11.49	14.12
MS	3190	258	20.19	10.47
MT	1989	154	10.51	8.09
NC	28,463	253	16.98	11.59
ND	1031	249	13.29	16.68
NE	3585	226	11.72	12.91
NH	3276	235	14.35	8.79
NJ	28,398	249	15.09	10.26
NM	3145	254	17.23	15.36
NV	16,085	241	13.62	11.78
NY	85,085	248	17.15	8.13
OH	33,013	235	17.67	11.51
OK	6270	260	14.64	14.19
OR	16,352	225	19.57	6.80
PA	40,430	232	14.82	7.38
RI	3377	237	13.86	9.30
SC	13,075	245	16.98	12.02
SD	1347	230	13.14	12.69
TN	17,085	257	16.89	11.29
TX	69,337	260	14.47	11.09
UT	4530	260	14.59	10.40
VA	22,677	245	14.72	14.44
VT	1936	247	15.39	11.05
WA	22,275	232	13.82	13.95
WI	13,886	229	12.00	11.21
WV	2279	270	12.11	19.53
WY	748	237	8.96	14.17

Table S2. Census tract level food environment characteristics and health outcomes (linear regression).

Census Tract Characteristics	Prevalence of Obesity			Prevalence of Diabetes			Prevalence of Hypertension		
	%	SE	*p*-value	%	SE	*p*-value	%	SE	*p*-value
**N = 18,504** [a]									
standardized mean calories [b]	0.18	0.04	<0.001 *	0.03	0.02	0.095	0.15	0.03	<0.001 *
standardized % healthy food [b]	−0.26	0.04	<0.001 *	−0.03	0.02	0.076	−0.10	0.04	0.008 *
standardized % fast food [b]	0.16	0.03	<0.001 *	0.02	0.01	0.073	0.12	0.03	<0.001 *

a. Census tract that have more than 10 tweets collected are included.
b. Twitter-derived food environment characteristics were standardized to have a mean of 0 and standard deviation of 1. Adjusted linear regression models were run for each outcome separately. Model controlled for census tract level demographics including population density, percent of 65 or older, percent of male, percent black, percent Hispanic, urban or rural area, percent relatives besides spouse and children living in households, percent unmarried cohabitating adults, household size, percent owner-occupied housing, and income inequality. Data Source: American Community Survey, CDC 500 cities.

Table S3. Twitter-derived food environment characteristics and sex-specific obesity rates, county level.

**Table 1a: Food environment characteristics and male obesity rate**				**Table 1b: Food environment characteristics and female obesity rate**			
	**%**	**SE**	***p*-value**		**%**	**SE**	***p*-value**
(Intercept)	31.22	0.07	<0.001	(Intercept)	29.47	0.10	<0.001
standardized mean calories [a]	0.32	0.07	<0.001	standardized mean calories [a]	0.42	0.10	<0.001
**Table 2a: Healthy Food Mean, Men**				**Table 2b: Healthy Food Mean, Women**			
	**%**	**SE**	***p*-value**		**%**	**SE**	***p*-value**
(Intercept)	31.22	0.07	<0.001	(Intercept)	29.47	0.10	<0.001
standardized % healthy food [a]	0.08	0.07	0.28	standardized % healthy food [a]	0.21	0.10	0.03
**Table 3a: Fast Food Mean, Men**				**Table 3b: Fast Food Mean, Women**			
	**%**	**SE**	***p*-value**		**%**	**SE**	***p*-value**
(Intercept)	31.22	0.07	<0.001	(Intercept)	29.47	0.10	<0.001
standardized % fast food [a]	0.13	0.07	0.07	standardized % fast food [a]	0.24	0.10	0.01

N = 2841 Counties included in both male- and female specific models, [a]Twitter-derived food environment characteristics were standardized to have a mean of 0 and standard deviation of 1. Adjusted linear regression models were run for male- and female-specific obesity rates separately. Model controlled for census tract level demographics including population density, percent of 65 or older, percent of male, percent black, percent Hispanic, urban or rural area, percent relatives besides spouse and children living in households, percent unmarried cohabitating adults, household size, percent owner-occupied housing, and income inequality. Data Source: American Community Survey, CDC 500 cities. The Chow test assessing differences in coefficients for Twitter-derived variables in the separate regression models for male- and female-specific obesity rates was non-significant for all Twitter-derived food variables, suggesting similar strength of associations between Twitter-derived variables and obesity rate for men vs. women.
